# Enhanced impact-resistance of aeronautical quasi-isotropic composite plates through diffused water molecules in epoxy

**DOI:** 10.1038/s41598-021-81443-w

**Published:** 2021-01-19

**Authors:** Furqan Ahmad, Fethi Abbassi, Mazhar Ul-Islam, Frédéric Jacquemin, Jung-Wuk Hong

**Affiliations:** 1 Department of Mechanical and Mechatronics Engineering, Dhofar University, Salalah, Oman; 2 College of Engineering and Technology, American University of the Middle East, Egaila, Kuwait; 3 Department of Chemical Engineering, Dhofar University, Salalah, Oman; 4 Research Institute in Civil and Mechanical Engineering, University of Nantes, Nantes, France; 5 Department of Civil and Environmental Engineering, Korean Advanced Institute of Science and Technology (KAIST), Daejeon, Korea

**Keywords:** Engineering, Materials science

## Abstract

In order to elucidate the hygroscopic effects on impact-resistance of carbon fiber/epoxy quasi-isotropic composite plates, low-velocity impact tests are conducted on dry and hygroscopically conditioned plates, respectively, under identical configurations. For the impact tests, plates were immersed in the hot water at 80 °C to absorb a different amount of moisture content (MC). Experimental results reveal that the presence of the MC plays a pivotal role by improving the impact-resistance of composite plates. Plates with higher percentage of MC could behave elastically to a larger strain, yielding larger deflection under impact loading. From SEM fractographies, it is observed that small disbanding grows at the interface of epoxy and carbon fiber due to absorbed MC. After absorbing MC, most of impact energy is dissipated in hygroscopic conditioned composite plates through elastic deformation and overall less damage is induced in wet composite plates compare to the dry plate. We can postulate that the presence of MC increases the elastic limit as well as ductility of the epoxy by promoting chain segmental mobility of the polymer molecules, which eventually leads to the enhancement of the impact-resistance of wet quasi-isotropic composite plates in comparison with the dry plate.

## Introduction

With higher stiffness to weight ratio as well as the improved strength, compared to the conventional structural materials, the composite materials especially carbon fiber composites have been gaining widespread use in many manufacturing industrial areas and are extensively used in the aerospace industry for aircrafts structural components such as wing flaps, engine cowlings, and vertical tail fin stabilizers. More than 37 years ago, the Boeing Commercial Airplanes used carbon fiber composite to manufacture the spoilers of the Boeing 737, a short-to-medium range twinjet narrow-body airline. Composite materials that have less maintenance cost and lighter in weight are replacing the alloy structure in the airplane industry. Boeing Commercial Airplanes company is using almost 50% composite material by weight for the manufacturing of Boeing 787 Dreamliner airplane^1,2^.

During the service life, composites such as aircraft outer bodies are exposed to different hygro-thermal conditions including changes in humidity and temperature. It has been reported that these different environmental conditions might cause degradation of mechanical properties by weakening the interfacial bonding between fiber and matrix and also cause some changes in the chemical properties of composite materials^3,4^. These hygro-thermal effects make this study crucial. In literature, only a few published research articles on the effects of hygroscopic conditions up to fully saturated condition on impact-resistance of quasi-isotropic composite materials are available. Zhong and Joshi^5^ performed impact tests on the normal and staggered lay-up composite structures which were exposed to hygroscopic condition and observed that impact resistance of wet composite structures increased. Zhou and Zhong^6^ checked the hygro-thermal effects on the properties of glass-vinyl ester composite which were exposed to freeze–thaw cycling. Then they performed the compression tests on the composites and observed degradation of compression properties. Mokhtar et al.^7^ studied the influence of aging on the impact damage of three different lay-ups of carbon fiber/epoxy composite plates and found that morphology of the impact damage in composite plates was significantly influenced by the aging process. Aoki et al.^8^ investigate the combined effects of thermal environmental conditions and moisture absorption on the compression of carbon fiber composite plates. They found that due to the presence of moisture the compression of the composite plate decreased slightly at 149 °C and significantly at 177 °C. Lu et al.^9^ investigate the effects of thermal conditions on the impact resistance of a unidirectional carbon fiber composite plate. The behavior of dry and hygroscopically conditioned composite materials under impact loading has major concern because impact load can cause different damages in composite materials such as matrix cracking, delamination between two adjusted layers, fiber and matrix interfacial disbanding, and breakage of fiber^10,11^. To understand the behavior of thin composite plate under dynamic loading such as impact loading, Zhao^12^ proposed a dimensionless damage number. Zhao’s dimensionless damage number depends on the geometry of the composite sample, impact loading condition, and material mechanical properties. In case of large value of Zhao’s dimensionless number, out-of-plane deformation or failure in the composite structure subjected to impact loading must be consider. During manufacturing as well as maintenance and repair, composite structures might experience the low-velocity impact load by dropping any tool, which causes damage in the structures and ultimately leads to the failure of structures^13,14^.

In the literature, no article is available on the effects of hygroscopic conditions (up to fully saturated condition) on the impact-resistance of quasi-isotropic composite materials due to their complex structure. Therefore, in this study, we investigate the effects of hygroscopic conditions up to fully saturated condition on the impact-resistance of carbon fiber/epoxy quasi-isotropic composite plate [0°/45°/90°/− 45°]_s_ under low-velocity impact loading. The main objectives of this study are to discern the relationship between the hygroscopic condition and impact-resistance of quasi-isotropic composite plate and to understand the mechanism by which MC changes the impact properties of the composite material. To achieve the main objective, low-velocity impact tests were performed using an impact testing machine on five different MC conditions: dry plate 0% (*Q*_1_), 0.75% (*Q*_2_), 1.0% (*Q*_3_), 1.1% (*Q*_4_), and fully saturated plate 1.36% (*Q*_5_). Composite plates were immersed in the distilled water to absorb the MC, and the temperature of the water was maintained at 80 °C to accelerate the absorption process. Fully saturated condition of the quasi-isotropic carbon fiber composite plate cannot be easily achieved because a long period of time (almost one and half year) is required, and consequently, data about the impact-resistance of fully saturated quasi-isotropic composite plates are rare in literature. Plates with a higher percentage of MC could behave elastically up to the higher level of strain and show larger deflection under impact loading. Wet quasi-isotropic composite plates show better impact-resistance in comparison with the dry plate due to presence of absorbed MC, which improves the ductility of the epoxy by promoting chain segmental mobility of the polymer molecules.

## Results

### Moisture absorption behavior and its effects

It is well known that the carbon fibers absorb a neglectable amount of water compared to the matrix (epoxy) in a humid environment. When the carbon fiber composite plate is immersed in the water, the water molecules do not diffuse at the surface of the plate and inside the plate because the carbon fibers change the diffusion path of the water molecules and hindering straight diffusion. Compared to any other stacking sequence, the quasi-isotropic plate shows maximum resistance to water molecules to diffuse inside the plate^4^.

**Figure 1:**
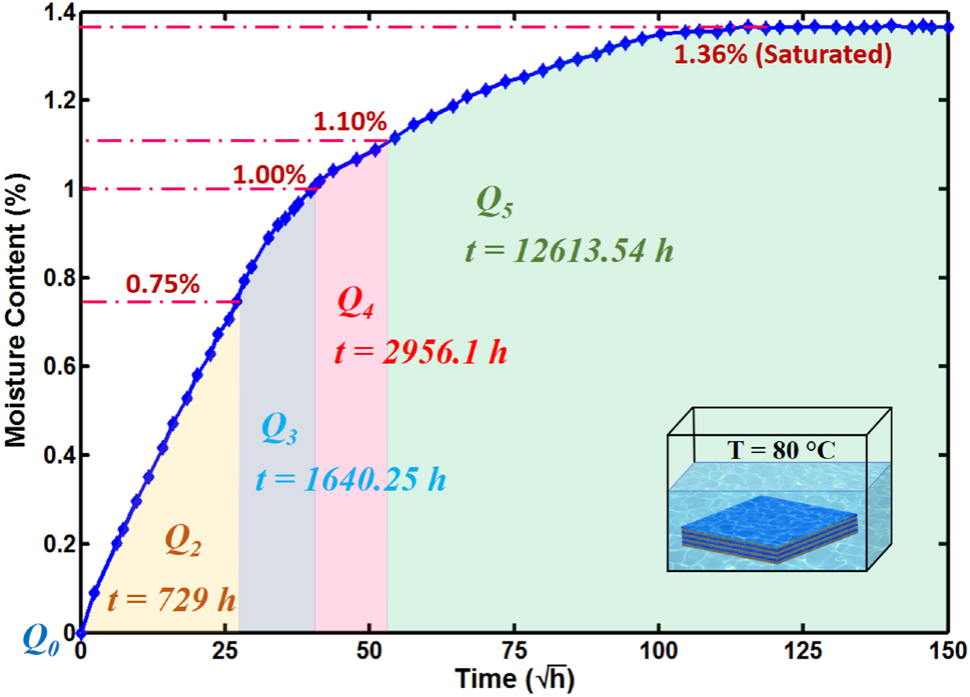
Water absorption behavior of quasi-isotropic composite plate at 80 °C.

**Figure 2:**
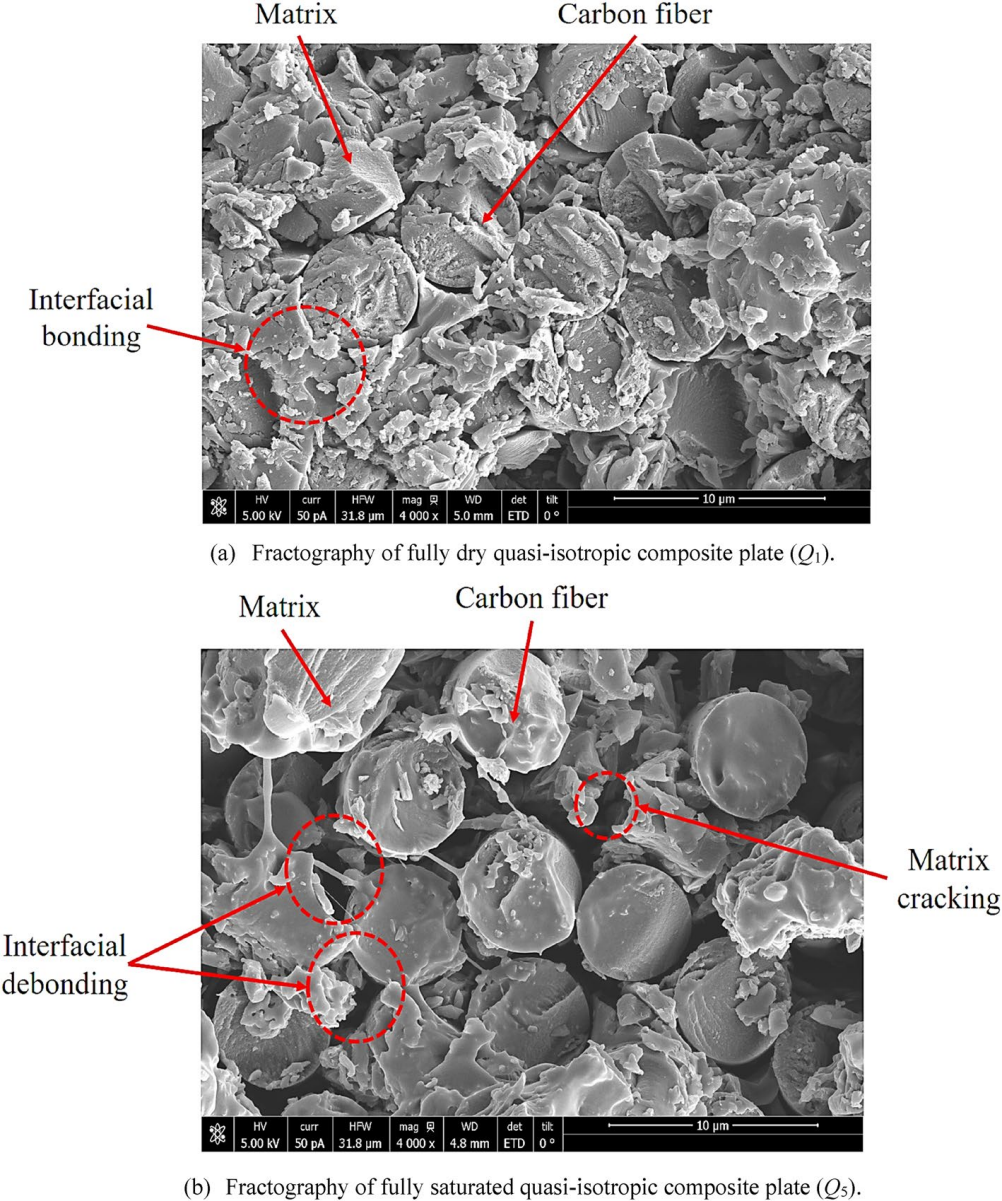
SEM fractographies for (**a**) fully dry and (**b**) fully saturation quasi-isotropic composite plates.

For each MC condition, weights of three dry specimens were measured, and then those specimens were immersed in the hot water at 80 °C. All plates were dipped completely into the water, and their average dry and wet weights for different MC conditions are listed in Table 1. From the moisture absorption curve in Fig. 1, it is observed that the dry plate absorbs water quickly and the curve raises linearly up until it absorbed almost 65% of its final saturated MC level. After that, the water absorption rate becomes very slow and the curve converges to the point of saturation. The composite plate absorbs the water molecules of 0.75% of its weight in the time period of 729 h and then took a long time of 1640.25 h to reach MC level of 1.0%. The 1.1% MC level, which is the fourth condition in this study, was achieved in the time period of 2956.1 h. To achieve a fully saturated level which is 1.36%, we need the immersion time of 12,613.54 h. To analyze the hygroscopic effects on the strength of the interfacial bond between the reinforced carbon fibers and epoxy matrix SEM, fractographies were performed for the fully dry and fully saturated plates as shown in Fig. 2. For the completely dry composite plate, a tight interfacial bonding between the carbon fiber and epoxy is seen clearly in Fig. 2a. When composite plates are immersed in the hot water, the carbon fibers do not absorb the water, and the moisture expansion coefficient is almost zero while the epoxy matrix has a high moisture expansion coefficient and absorbs almost all of the water molecules. Due to absorbing water molecules, the epoxy matrix swelled up and caused interfacial debonding with the carbon fibers as shown in Fig. 2b.

**Table 1:** Moisture absorption related parameters for quasi-isotropic composite plates.

Specimen ID	Number of specimens	*W*_d_ (g)	*W*_w_ (g)	*M* (%)	Immersion time (h)	*D*_t_ (mm^2^/h)
*Q*_1_	3	37.2718	0.0000	0.00	0.0	2.23 × 10^–4^
*Q*_2_	3	37.2841	37.5637	0.75	729.54	
*Q*_3_	3	37.2799	37.6527	1.00	1640.25	
*Q*_4_	3	37.2572	37.6670	1.10	2956.10	
*Q*_5_	3	37.2684	37.7753	1.36	12,613.54

### Low-velocity impact behavior

To investigate the hygroscopic effect on the quasi-isotropic composite plates, all experimental low-velocity impact tests were performed using identical impact energy. To understand completely the hygroscopic effect, a range of experimental tests from fully dry to fully saturated conditions were performed. Firstly, impact tests were performed for the fully dry composite plate (*Q*_1_) and its experimental results were set as a reference. Later, four more impact tests were performed for different absorbed MC conditions (percentage by weight of composite plates) such as 0.75% (*Q*_2_), 1.0% (*Q*_3_), 1.1% (*Q*_4_), and fully saturated 1.36% (*Q*_5_). Fully saturated condition of the quasi-isotropic carbon fiber composite plate cannot be achieved easily as a long period of time (almost one and half year) is required.

**Figure 3:**
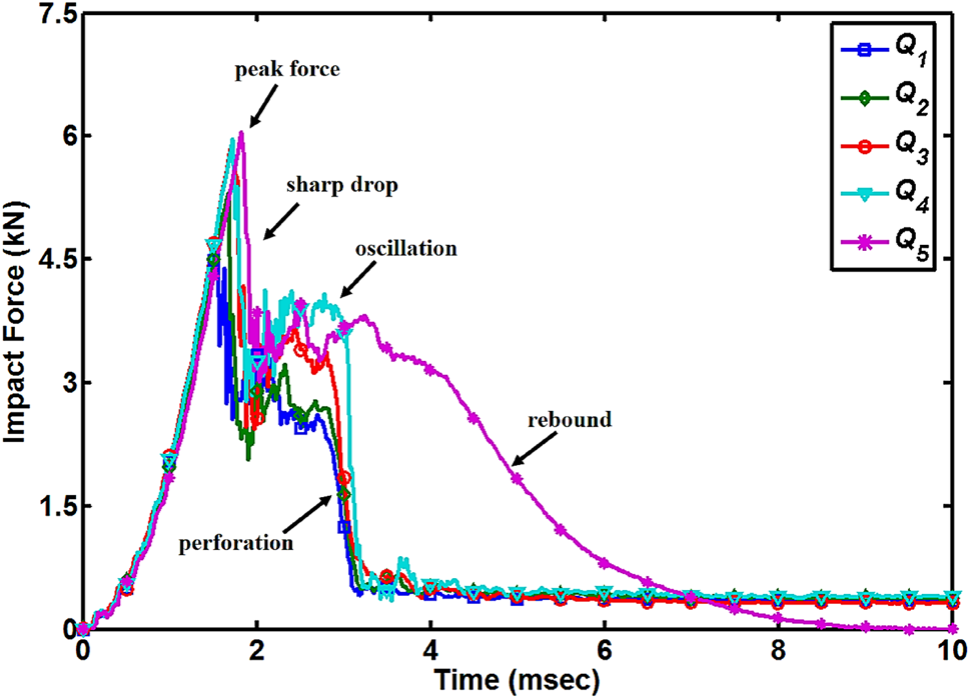
Impact force versus time diagram for all quasi-isotropic composite plates.

When the hemispherical shaped impactor contacts on the surfaces of the composite plates, the responses of those composite plates were obtained in form of voltage versus time graphs using a load cell, and later these data were converted to force versus time graphs. Complete depiction of the contact between hemispherical shape impactor and composite plates can be studied from the force versus time graph which gives the details of incipient damage, layer-by-layer damage growth, and overall changes in the impact-resistance of the composite plates. Force versus time graphs for all the quasi-isotropic composite plates is shown in Fig. 3. During the experimental tests, as the impactor hit the top layer of 0˚ of the composite plates, the impact damages in form of matrix cracking at a micro-level and delamination between the adjacent layers started and these damages can be observed as an oscillation at the beginning of the force curves. These oscillations in the force curves are referred to as Hertzian failure of the composite materials. All the curves moved toward their peak force values with the almost same slope, which indicates the out-of-plane stiffness of the quasi-isotropic composite plates does not affect. Initially, the majority of the impact load was carried by the carbon fibers which were not affected by the absorbed MC. All the curves reached their peak force values in a linear manner without any oscillations in a short period of time. After the peak force point, a sharp drop was observed which is the indication of rapid extension of damages. Through the thickness of the plates, damages occurred in the form of matrix cracking and fiber breakage which appears as large scale oscillations in the force curves. For the first four cases (*Q*_1_ to *Q*_4_), after the large scale oscillations, again a sharp drop happened which is the indication that all carbon fiber dominated layers of composite plates are broken and impactor is perforated through the plates. Small scale oscillations happened after the perforation due to the friction between the perforated plates and impactor surface during the downward movement of the impactor. For the fully saturated composite plate (*Q*_5_), after the large scale oscillations, a smooth drop of the curve was observed which is the indication that the impactor is rebounding back and no damage occurred in this rebound portion. Impact event characteristics changed because of the absorbed MC from the supercritical impact event for the *Q*_1_ composite plate to the critical impact event for the *Q*_5_ composite plate^15,16^. *Q*_5_ composite plate got the highest peak force value of 6.054 kN and *Q*_1_ composite plate showed the lowest peak force value of 4.651 kN which means due to absorbed MC the impact-resistance of the quasi-isotropic composite plates increased. The time corresponding to the peak force value also increased with the increase of the percentage of the absorbed MC, implying that the first significant damage of quasi-isotropic or occurrence of incipient damage is delayed by 0.275 ms for *Q*_5_ composite plate from the *Q*_1_ composite plate as shown in Fig. 3. The peak force values and their corresponding times for all specimens are shown in Table 2.

**Table 2:** Parametric comparisons for low-velocity impact tests for quasi-isotropic composite plates.

Specimen ID	Time at peak force (msce)	Peak force (kN)	*E*_ini_ (J)	*R*_*a*_ (%)	*E*_*res*_ (J)
*Q*_1_	1.550	4.651	8.438	35.647	13.409
*Q*_2_	1.675	5.307	10.148	42.873	12.219
*Q*_3_	1.700	5.853	11.105	46.916	11.933
*Q*_4_	1.725	5.970	11.685	49.368	11.870
*Q*_5_	1.825	6.054	12.008	50.729	11.667

**Figure 4:**
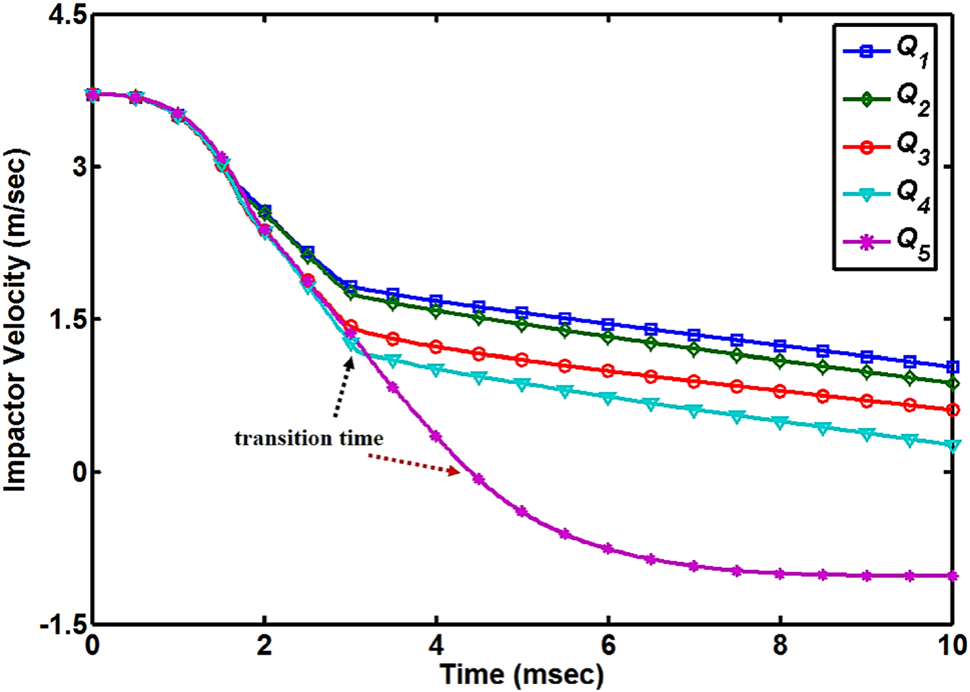
Impactor velocity versus time diagram for all quasi-isotropic composite plates.

**Figure 5:**
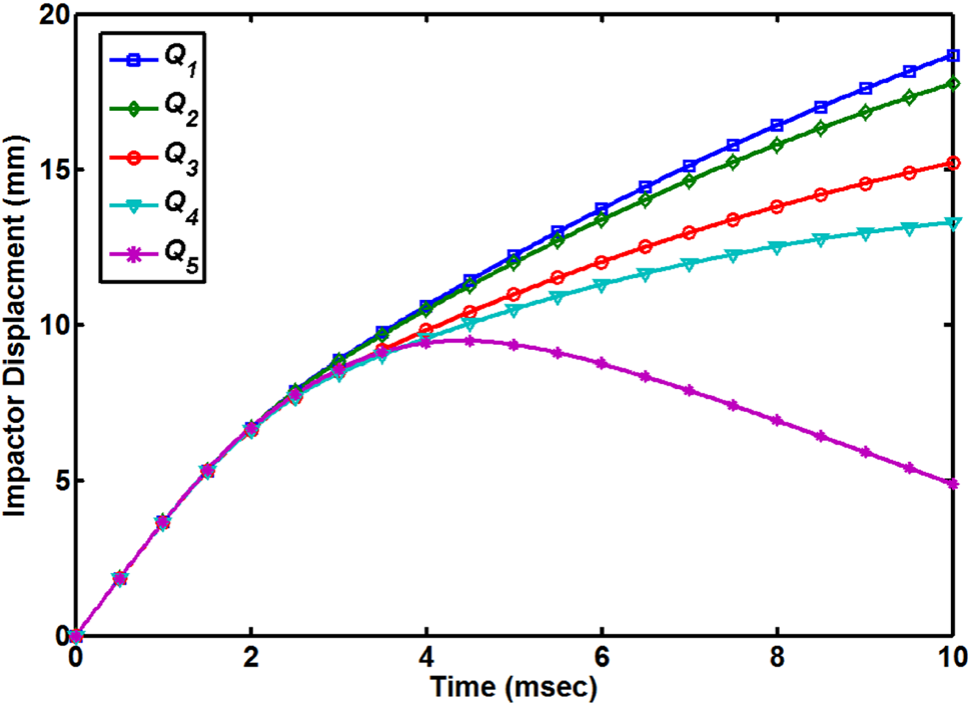
Impactor displacement versus time diagram for all quasi-isotropic composite plates.

**Figure 6:**
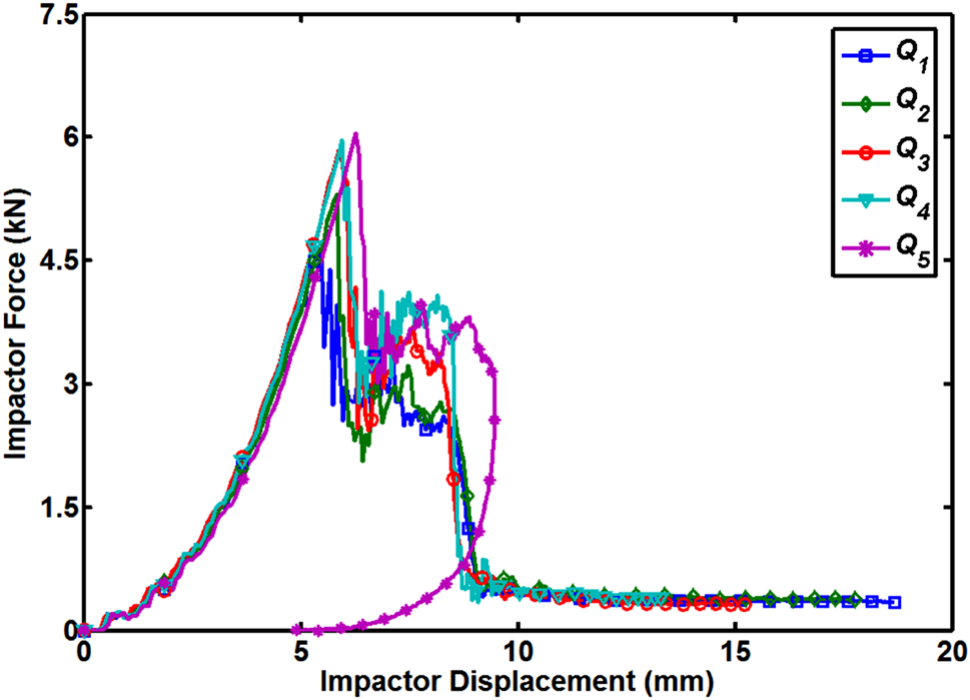
Impact force versus displacement diagram for all quasi-isotropic composite plates.

Kinematics formulas defined in following Eqs. (1) and (2) are used to extract the impactor velocity and displacement data from the impact force versus time graphs for the better understanding of the impact behavior of the dry and moisture absorbed quasi-isotropic composite plates under low-velocity impact loading.

1 $${v}_{t}={v}_{i}-\frac{1}{{m}_{i}}{\int }_{0}^{t}{F}_{i} dt$$

2 $${d}_{t}={d}_{i}+{\int }_{0}^{t}{v}_{t} dt$$

where *v*_t_ and *d*_t_ is the impactor velocity and displacement at time *t*, *v*_i_ and *d*_i_ is the initial velocity and displacement of the impactor just before contact with the top surface of the composite plate, *F*_i_ is the recorded force of the impact and *m*_i_ is the impactor load. The time-velocity curves for all the quasi-isotropic composite plates are shown in Fig. 4. For the first four cases (*Q*_1_ to *Q*_4_), the impactor velocity curves dropped quickly from their initial value of 3.71 m/s to the time at the second sharp drop in the time-force curves occurred which is corresponding to the failure of the composite layers and impactor is perforated through all layers. The time corresponding to this point is called transition time (showing with dotted black arrow) and after this point, the drop in the curves is small and linear which is the indication that the length of the impactor is passing slowly through the thickness of the composite plates. For the *Q*_5_ composite plate, the time-velocity curve dropped quickly from the initial velocity level and become zero at the transition time (showing with dotted red arrow) and after this point, the velocity of impactor become negative which mean the impactor started to rebound. The rebound of the impactor cannot be observed in the time-force curve so that’s why it is important to draw the impactor velocity graph for a better understanding of the impact behavior of the quasi-isotropic composite plates. With the increase in the percentage of MC, the transition time of composite plates increased. Time-impactor displacement curves for all five MC conditions are shown in Fig. 5. During the impact tests, the impactor kept moving without any barrier on it and displacement curves for the first four (*Q*_1_ to *Q*_4_) cases increased. *Q*_1_ composite plate shows less resistance to the impactor and got the maximum displacement value of 18.68 mm at a time of 10 ms and at the same time *Q*_4_ composite plate shows less displacement level of 13.29 mm. Impactor does not perforate through the *Q*_5_ composite plate and when at the point of impact maximum deflection of the plate occurred, the impactor achieved its maximum displacement of 9.47 mm and after the transition time impactor started to rebound back to zero displacement position (original level) where it will be completely detached from the top layer of 0˚. The displacement versus force curves of all the five MC conditioned specimens are shown in Fig. 6. This rebound back of the hemispherical head impactor can also be observed in Fig. 6 where displacement versus force curve started to fold with decreasing displacement of the impactor. As impactor perforated through the first four quasi-isotropic composite plates (*Q*_1_ to *Q*_4_), there is no fold of displacement versus force curves observed for these specimens. Displacement of the impactor decrease with the increase of MC and impactor rebound back for *Q*_5_ specimens indicated that the impact-resistance of quasi-isotropic composite plates improved under hygroscopic effect.

**Figure 7:**
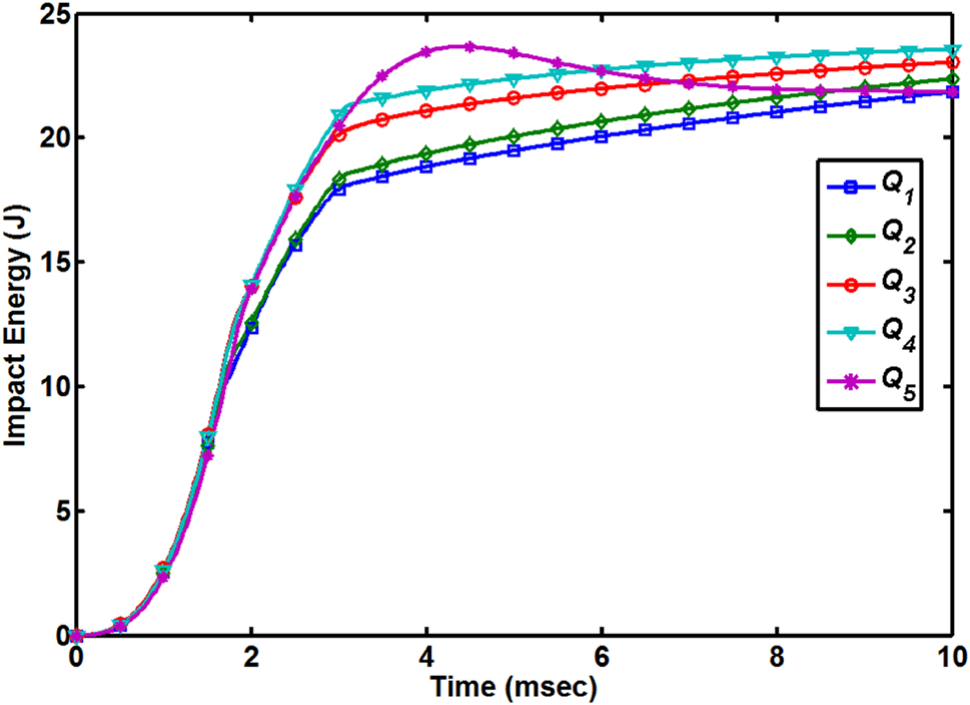
Absorbed energy versus time diagram for all quasi-isotropic composite plates.

To analysis the hygroscopic effect on the impact-resistance of the quasi-isotropic composite plates, impact tests were performed under the same level of impact energy of 23.67 J. Energy versus time graphs for all the quasi-isotropic composite plates are shown in Fig. 7. During the impact tests, energy absorbed by the quasi-isotropic composite plates from the energy applied by the impactor was calculated as

3 $${E}_{i}=\frac{{m}_{i}}{2}{{v}_{i}}^{2}$$

4 $${E}_{a}=\frac{{m}_{i}}{2}{{v}_{i}}^{2}-\frac{{m}_{i}}{2}{{v}_{t}}^{2}$$

where *E*_i_ is the initial or applied energy and *E*_*a*_ is the absorbed energy. In Fig. 7, energy curves can be categorized into two cases: impactor perforated through the plates (for first four cases *Q*_1_ to *Q*_4_) and impactor penetrated the plate (for *Q*_5_ composite plate) and rebound back. Initially, the rate to absorb the impact energy was very low for all the composite plates which was attributed to the initiation of damage in form of dent on the top surface and crack along the thickness direction at the point of impact. After that between the time period of 1 to 2 ms, the composite plates absorbed the impact energy during the deflection of the composite plates and the slopes of all energy curves were increased quickly and internal damages were produced in composite plates. Energy curves for the first four cases (*Q*_1_ to *Q*_4_) rise with a large slope progressively until the maximum deflection of the composite plates reached and internal damages started in the plates. When the impactor perforated through these composite plates, maximum energy was absorbed by the plates after that the energy curves appeared to further increase because the later surface of the impactor still has contact with the circular edges of the perforated plates. For the *Q*_5_ composite plate, after reaching the peak point the energy curve starts decreasing progressively which is an indication that impactor has started the rebound. It is reasonable to state that when the impactor and plate detached completely, any further absorption of the energy did not occur.

Force versus time curves for all the composite plates can be divided into two distinct regions: a region before the peak force can be referred to as fracture initiation region and region after the peak force point is referred to as the fracture propagation region. The energy absorbed by the composite plate up to peak force point and required for fracture ignition can be defined as initiation energy (*E*_ini_). As energy curves are categorized in two cases so it is reasonable to compare the initiation energies for all the plates. *E*_ini_ values for all the five cases (*Q*_1_ to *Q*_5_) are listed in Table 2, Q5 composite plate has the highest initiation energy and the lowest is for the *Q*_1_ composite plate. With the increase of MC, the value of the initiation energy increased.

To check how much energy was absorbed by the composite plates to reach the peak force point, an absorbed energy ratio (*R*_*a*_ %) is introduced to compare the ratio of initiation energy over the energy applied by the impactor as

5 $${R}_{a} (\mathrm{\%})= \frac{{E}_{ini}}{{E}_{applied}}\times 100$$

and those values are listed in Table 2 for all composite plates. As *Q*_5_ composite plate has the highest peak force point and got the absorbed energy ratio of 50.729% and lowest ratio (35.647%) is for the *Q*_1_ composite plate.

After the peak force point or initiation energy, energy curves further increased and reached their maximum point (*E*_max_) which is corresponding to the perforation of the composite plates (*Q*_1_ to *Q*_4_) and the maximum deflection of the fully saturated composite plate (*Q*_5_). It is reasonable to assume that energy absorbed by composite plates from initiation to maximum energy points was utilized to produce the major damages such as delamination and energy absorbed between these two points is referred to as residual energy (*E*_res_), defined in the following Eq. (6). The initiation energy, maximum energy, and residual energy for composite plates with five different MC conditions are listed in Table 2.

6 $${E}_{res}={E}_{max}-{E}_{ini}$$

From Table 2 it is also clear that *Q*_5_ composite plate has maximum initiation energy (12.008 J) and utilized more than 51% of applied energy to reach the peak force point and reaming 49% was used to produce the damages. *Q*_1_ composite plate reached more quickly to peak force point by using the 8.438 J initiation energy and utilized almost 35% of applied energy to reach this point and the remaining 65% of applied energy was used to produce the damages.

### Impact damage analysis

**Figure 8:**
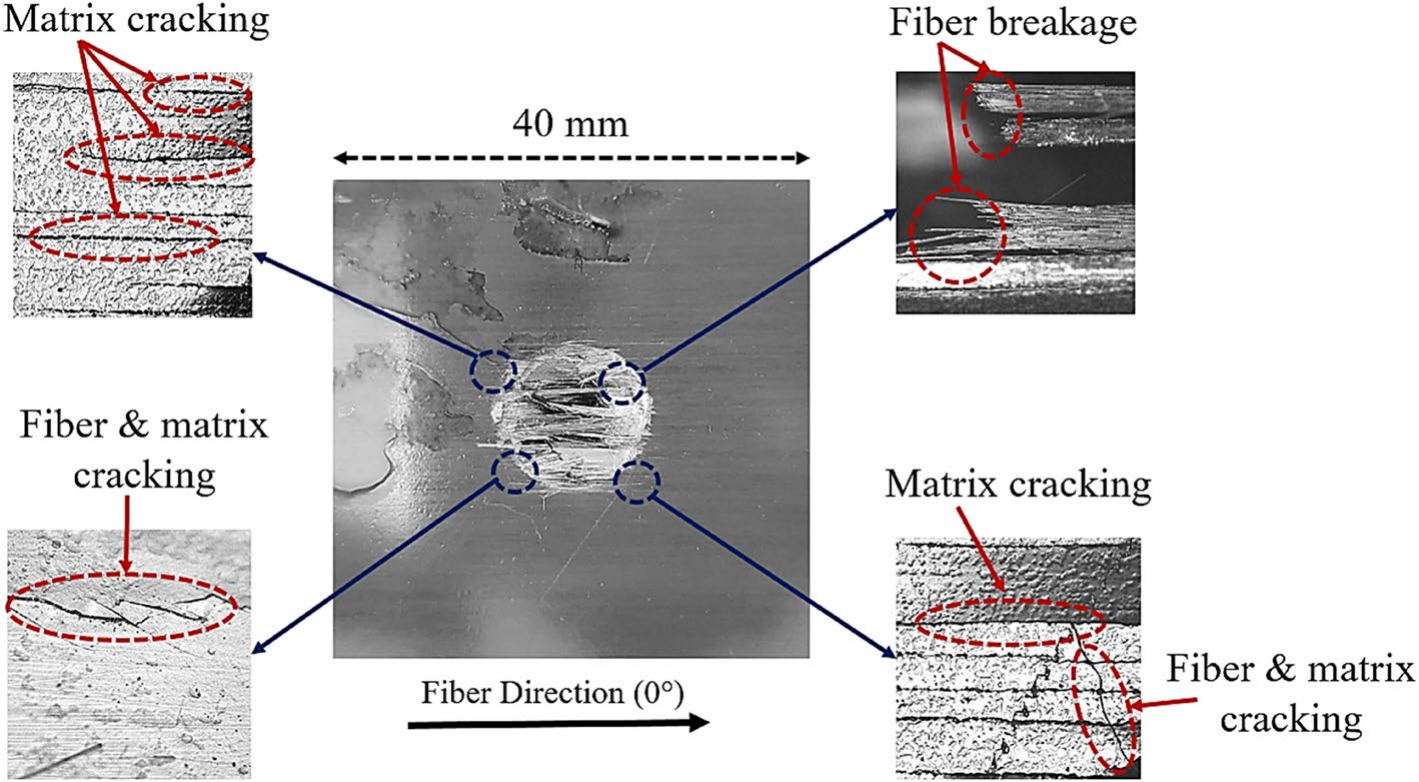
Damages on the top face of fully dry composite plate (*Q*_1_).

**Figure 9:**
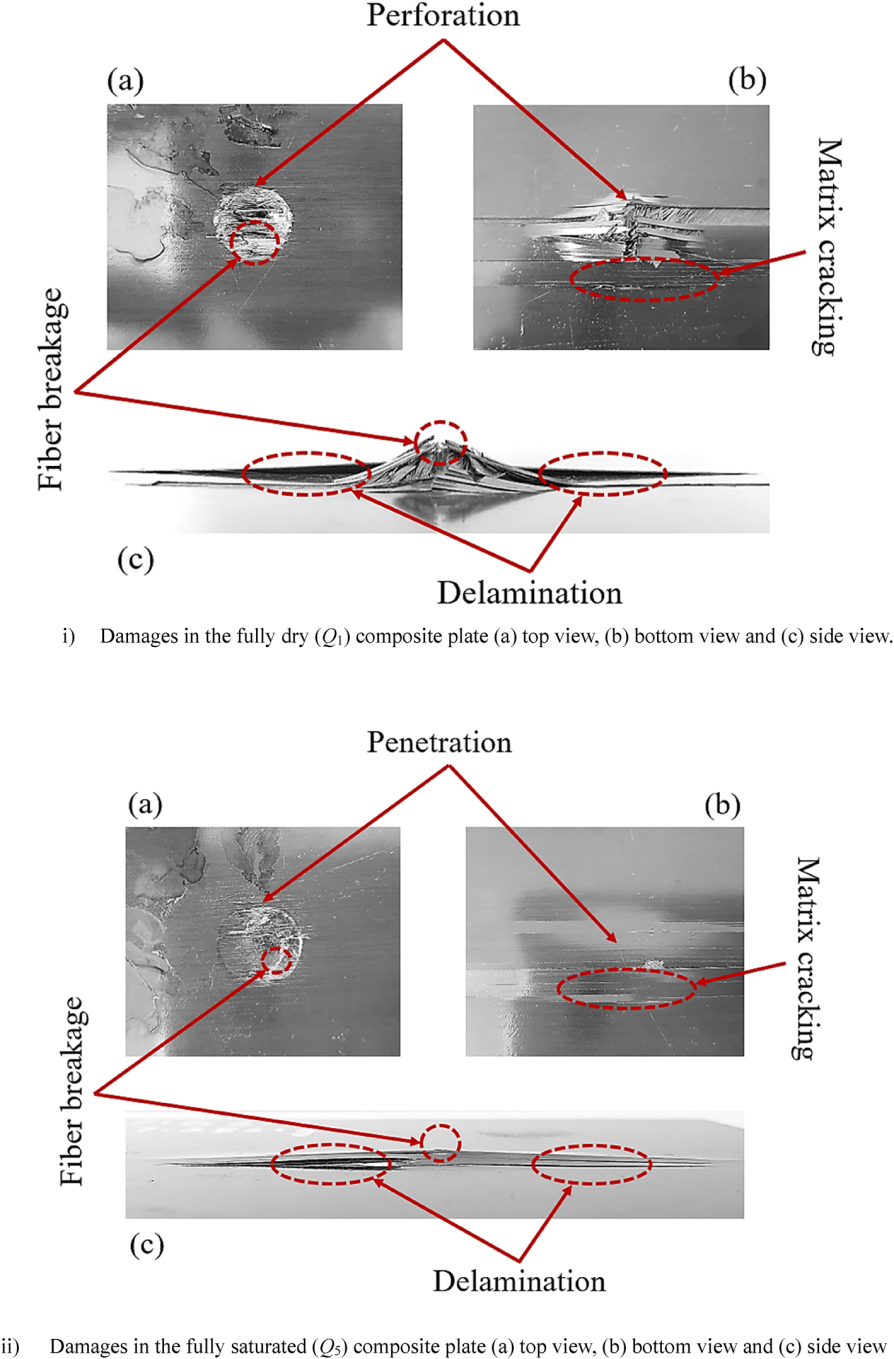
Different views of damages in (i) fully dry (*Q*_1_) and (ii) fully saturated (*Q*_5_) composite plates.

Whenever an object falls on the composite structures, a complicated damage mechanism consists of different failure modes are created. Matrix cracking, fiber, and matrix breakage, fiber and matrix interfacial debonding, inter-laminar cracking, and delamination are the major damage modes that will occur under impact loading and these modes also interact with each other^17,18^. Damage in the form of matrix failure initiates in the composite materials when the applied impact energy surpassed the threshold level. Compared to the matrix, more impact energy is required to fail the fibers in the composite materials. It is reasonable to say that strength and impact impact-resistance of the composite materials mainly depend more on matrix strength than the fiber strength. For all five cases (*Q*_1_ to *Q*_5_), when impactor dropped on the top face of the composite plates, fiber breakage, fiber cracking, and matrix cracking were observed as shown in Fig. 8 for *Q*_1_ composite plate. At the point of impact, fibers were broken and the matrix was failed due to indentation effects which cause local high stresses. The top and bottom faces of the *Q*_1_ composite plate are shown in Fig. 9i. As the impactor perforated through this plate, circular shape damage was observed on the front face and underneath the impact point between the last two layers delamination was occurred. Impactor did not perforate through the *Q*_5_ composite plate but create a large dent on the top face as shown in Fig. 9ii. Different damage modes such as matrix and fiber crushing, matrix cracking, and fibers breakage took place underneath of impact point on the top face, and delamination was observed on the back face. From Fig. 9 it can be observed that more damages occurred in the *Q*_1_ composite plate compare to the *Q*_5_ composite plate means impact-resistance of quasi-isotropic composite plates improved with the presence of absorbed MC.

## Discussion

**Figure 10:**
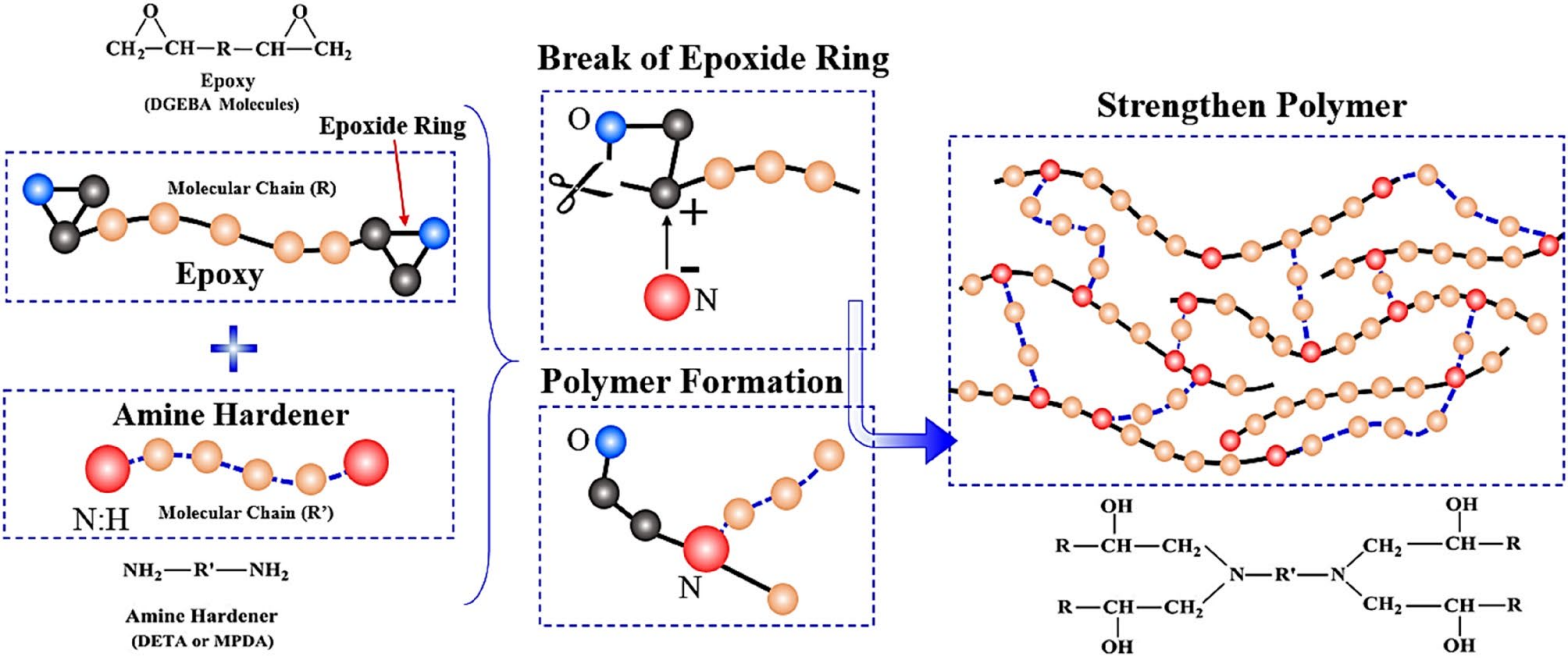
Schematic representation of reactants and formation of crosslinks in epoxy resin.

**Figure 11:**
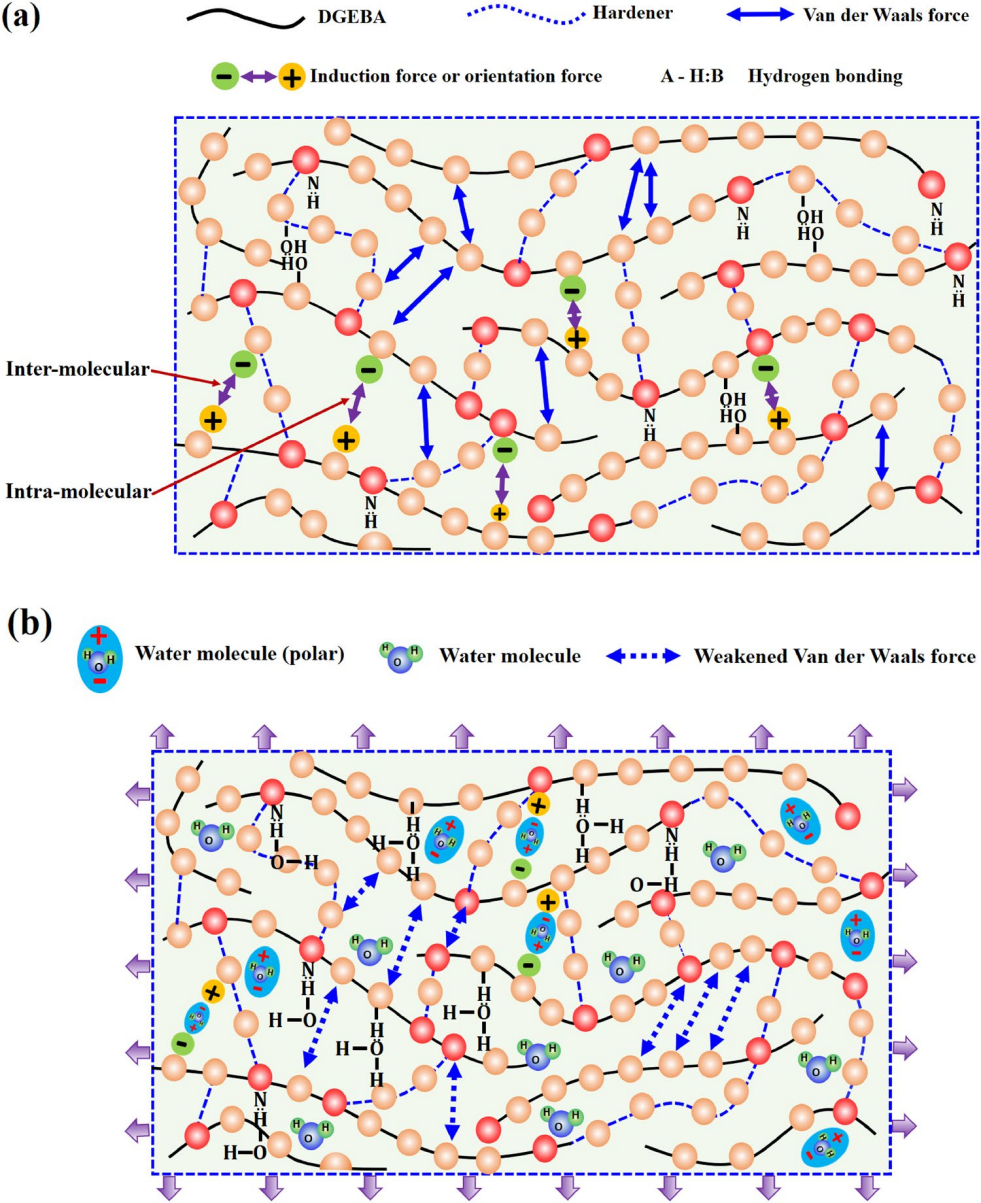
Schematic diagram of the (**a**) Van der Waals force, induction force or orientation force, and hydrogen bonding of epoxy molecules before absorbing the moisture content and (**b**) interaction between the moisture content and epoxy, weakened Van der Waals force, and disruption of hydrogen bonding of epoxy molecules after absorbing moisture.

Based on the low-velocity impact test results, it can be concluded that the elastic deformation capacity of the composite plates was improved by absorbing the MC. From the SEM fractography (Fig. 2) of fully dry (*Q*_1_) and saturated (*Q*_5_) composite plates, it was observed that absorbed MC had minimal effects on the carbon fibers and had some effect on the interfacial bond between fiber and matrix. Therefore, it can be postulated that absorbed MC affects the matrix resin and improve the elastic deformation capacity of composite plates^5,6^. Compare to the water molecules, the molecules of the cured epoxy are large in size. That’s why water molecules can diffuse into the epoxy resin network easily and reside in the vacancies between the chains of the epoxy molecules and cause an increase in the overall thickness of the composite plate and expansion of its volume. The covalent bond force between the small molecules are normally greater in magnitude (one to two times) compare to the Van der Waals force. Polymers molecules have long structural chains and greater in number which characterized them huge molar mass structures. The inter-molecular force (Van der Waals force) of the long structural chain of the polymer molecule is comparatively high than the covalent bond force. Figure 10 shows the formation of the crosslinked polymers from reactants (epoxy and amine hardener). During the curing cycle, each amine group of the hardener molecule (Diethylenetriamine (DETA) or Meta Phenylene Diamine (MPDA) hardener) have two active atoms of the hydrogen which react with the epoxy (Diglycidyl Ether of Bisphenol A (DGEBA) molecule) molecule and form the long-chain structure of the crosslinked polymers and inter-molecular force became stronger than the covalent bond force^5,6^. This strong inter-molecular force leads to the brittle behavior of the fully dried composite material under the impact loading. The inter-molecular force in the long structure of the polymer molecule is classified into two parts, Van der Waals force, and hydrogen bonding. Van der Waals force is further divided into three parts such as orientation force, induction force, and dispersion forces as shown in Fig. 11a. There are two aspects by which the intermolecular force of polymer molecules can be weakened by the interaction between the epoxy resin and water molecules (Fig. 11b). Firstly, the Van der Waals force between the polymer molecules is effective up to a certain distance and beyond this distance starts to become weaker. As large-sized water molecules diffused in the long structure of the polymer molecules, the distance between the polymer molecules increased and the Van der Waals force becomes weak. Induction and orientation forces are mainly depending upon the polar groups of the polymer molecules and water molecules are also polar molecules as shown in Fig. 11b. So, the water molecules make coupling with the polymer molecules and weaken the induction and orientation forces. Secondly, the amine and hydroxyl groups of the epoxy resin are ready to make the hydrogen bonding. The diffused water molecules can also create hydrogen bonding with epoxy molecular chains and reduced the number of hydrogen bonding of the epoxy resin. Due to the expansion of the volume of the epoxy resin, some hydrogen bonds may also destroy. Therefore, hydrogen bonding became weak due to the presence of the absorbed MC. It can be concluded that overall inter-molecular attractive forces of the long-chain epoxy resin were became weakened due to the presence of the absorbed MCs by hindering the Van der Waals forces and disrupting the hydrogen bonding of the epoxy resin. The behavior of the hygroscopic composite plates changed from brittle to ductile because of promotion in the movement of the various epoxy resin molecules and other molecules against each other.

As shown in Fig. 3, time to reach the peak force point is a delay with the increase of MC and the occurrence of the first significant damage or a sharp drop in the time-force curves also delayed. This delay in the first significant damage can also be observed in Fig. 6 which shows that damages in *Q*_5_ composite plate started at the larger displacement of the impactor corresponding to higher strain level compare to *Q*_1_ composite plate. This delay in the occurrence of the damages shows that the ductility of matrix resin improved due to the presence of MC. The improved ductility would benefit the laminate by hindering the initiation of inter-laminar cracks and matrix cracking but debonding and fiber breakage will not be delayed. Improved ductility of the matrix resin, increased the strain to first significant failure and hygroscopic conditioned quasi-isotropic composite plates preserved their structural integrity up to a large displacement of the impactor. The ductility of matrix resin increase with the increase of the percentage of MC.

## Conclusions

An experimental to investigation the hygroscopic effect on the impact-resistance of symmetric quasi-isotropic CFRP composite plates under low-velocity impact loading was presented in this study. From the current experimental study for different percentages of MC, the following conclusive remarks are drawn. Presence of the carbon fiber in quasi-isotropic composite plate cause hindering to the water molecules in straight diffusion by changing the diffusion path. Therefore, it took more than one and a half years to reach a fully saturated point. From the SEM images, it was observed that absorbed MC cause debonding between matrix resin and carbon fiber at their interfacial. From the low-velocity impact results, it was observed that with the increase of MC percentage the time to reach peak force point and peak force values increased. From the impactor velocity and displacement curves it was observed that impactor was perforated through the first four composite plates (*Q*_1_ to *Q*_4_) and impactor rebound for fully saturated composite plate (*Q*_5_). Initiation energies and absorbed energy ratios increase with the increase in the percentage of MC and residual energy decreases. A delay in the first significant damage was observed due to increase ductility of composite plates and damages in fully saturated (*Q*_5_) composite plate occurred at the larger displacement of the impactor compare to fully dry (*Q*_1_) composite plate. The overall size of damages on the top and bottom faces decreases with an increase of the percentage of MC. More damage was observed for the fully dry composite plate (*Q*_1_) compared to hygroscopic conditioned composite plates (*Q*_2_ to *Q*_5_). From the current study, it is reasonable to say that the presence of the MC improved the ductility of quasi-isotropic composite plates and eventually enhanced the impact-resistance of the quasi-isotropic composite plates under low-velocity impact loading.

## Methods

### Composite material and specimens

Quasi-isotropic composite plates are fabricated from pre-impregnated unidirectional carbon fiber (T800/3900) tape manufactured by the Toray Industry, Inc. This tape consists of intermediate modulus carbon fiber (T800) and 350 ˚F cure-type toughened epoxy (3900). The material has a standard 55.5% fiber volume fraction. From the roll of carbon fiber unidirectional pre-impregnated tape, large pieces of 1000 mm × 1000 mm were cut at fiber angle of 0˚ and 45˚. Then, the pieces were laid-up to fabricated the symmetric quasi-isotropic composite plate [0°/45°/90°˚/− 45°]_s_ of eight plies. For the curing purpose, an autoclave with good control on curing parameters such as vacuum, heat-up rate, temperature, and pressure was used (the curing cycle was inlined with the parameters provided by the company). From the fabricated large panels, square plates of 125 mm × 125 mm with a nominal thickness of 1.5 mm were cut. The mechanical properties of a single lamina obtained from the company are listed in Table 3.

**Table 3:** Material properties of the single layer.

Material properties	Value
Young’s modulus in the fiber direction (*E*_*1*_)	142 GPa
Young’s modulus in the transverse direction (*E*_*2*_)	7.79 GPa
Poisson’s ratio (*v*_*12*_)	0.34
Poisson’s ratio (*v*_*23*_)	0.53
Shear modulus (*G*_*12*_)	4.0 GPa
Shear modulus (*G*_*23*_)	2.55 GPa
Tensile strength in the fiber direction (*X*_*T*_)	2251 MPa
Compressive strength in the fiber direction (*X*_*C*_)	1078 MPa
Tensile strength in the transverse direction (*Y*_*T*_)	58.47 MPa
Compressive strength in the transverse direction (*Y*_*C*_)	199.81 MPa
Shear strength (*S*_*12*_)	69.36 MPa
Density (*ρ*)	1550 kg/m^3^

### Experimental setup

To observe the water absorption behavior of the quasi-isotropic composite plate, an accelerated immersion water test was performed. A preconditioning procedure was performed by putting the composite plates in the oven at 50 °C to remove the residual MC and make them completely dry. Before putting plates to the refrigeration bath circulating machine, the dry weight (*W*_d_) of each plate was recorded with a precision electric weight scale with high-level of accuracy (± 0.0001 g) and later it will be used as a reference for each plate. The tub of the refrigeration bath circulating machine was filled with distilled water and a high temperature of 80 °C was set to accelerate the water transport process. This temperature is lower than the melting point of the quasi-isotropic composite plates and epoxy glass transition temperature (*T*_g_). *T*_g_ of epoxy in dry state is 204 °C and in the wet state is 166 °C according to the data sheet provided by the Toray Company. *T*_g_ of epoxy in dry and wet states are far away from the hot water temperatur of 80 °C. Composite plates were fully immersed in the water and taken out from the water and their wet weight (*W*_w_) was recorded after a specific interval of three days. Before measuring the wet water, the available water drops were completely removed from the surfaces of the plates to avoid the error in measurements. To avoid discontinuity in the water absorption process and any environmental effect, the weight was recorded in a short period of time less than 5 min. Based on the periodic *W*_w_ measurements, the percentage of water absorbed by the composite plates were measured using Eq. (7)

7 $$M(\%)=\frac{{W}_{w}-{W}_{d}}{{W}_{d}}X100$$

According to the ASTM standard^19^, if the aspect ratio (width/thickness) of the composite plate is smaller than 100, the diffusion of the water molecules will be Fickian diffusion. Therefore, the diffusion takes place through the thickness direction of the plate and the edge's effects can be neglected. In the current study, the aspect ratio of the quasi-isotropic composite plate is 83.33, which is smaller than the 100. Consequently, Fick’s diffusion law^20^ was used to calculate the water diffusion coefficients of the composite plate. The water diffusivity (*D*_t_) through the plate thickness direction is written as

8 $${D}_{t}=\pi {\left(\frac{{t}_{cp}}{4{MC}_{eq}}\right)}^{2}{\left(\frac{{MC}_{2}-{MC}_{1}}{\sqrt{{t}_{2}}-\sqrt{{t}_{1}}}\right)}^{2}$$

where *t*_*cp*_ is the composite plate thickness, *MC*_eq_ is the equilibrium percentage of the MC, and *MC*_1_ and *MC*_2_ are two different MC levels measured after the time intervals of *t*_1_ and *t*_2_, respectively.

**Figure 12:**
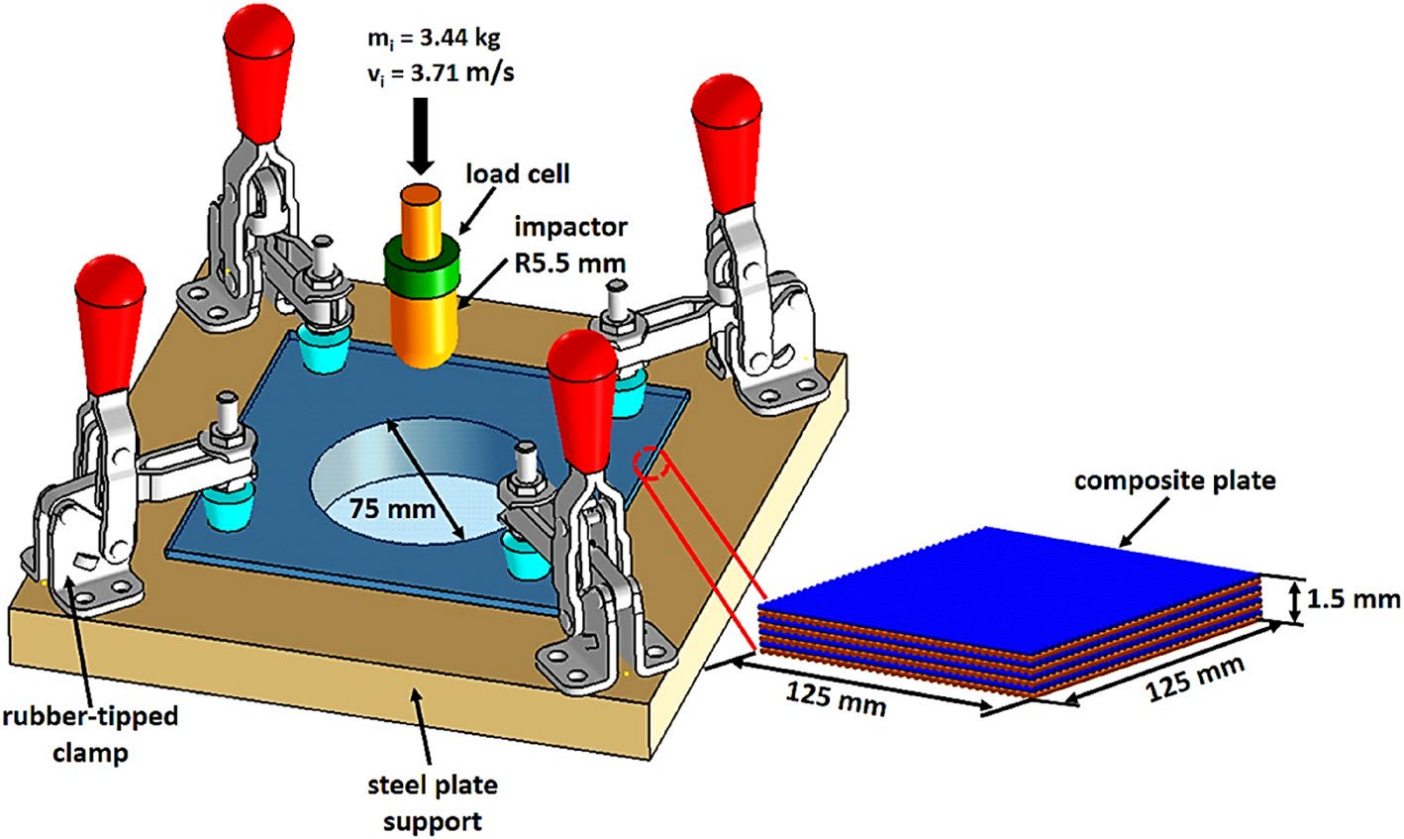
Schematic diagram low-velocity impact test for quasi-isotropic composite plates.

Vertical impact testing machine was used to perform the low-velocity impact tests on dry and hygroscopic conditioned quasi-isotropic composite plates. Schematic diagram of the complete low-velocity impact testing set up is shown in Fig. 12. The total mass of impactor (*m*_i_) including all add-on weights and load cell was 3.44 kg and 0.7 m height of impactor (*h*_i_) was selected corresponding to initial impact velocity (*v*_i_) of 3.71 m/s and provide the impact energy of 23.67 J. To analyze the hygroscopic effect, all the composite plates were tested under constant boundary conditions. Composite plates were put on the steel plate support that has a circular cut of 75 mm diameter at the center to allow perforation of impactor through the thickness of plates without any interference. To prevent in and out of the plane movement of the composite plate, the plates were clamped at four corners by the rubber-tipped clamps those applying the light clamping pressure. All this arrangement simulates the simply-supported boundary condition. For each hygroscopic condition, three impact tests were performed to avoid the experimental error and after impact test, composite plates were removed from the fixture with care for the post-impact damage analyses.
